# Editorial: Looking Beyond Pattern Recognition: Perturbations in Cellular Homeostasis and Metabolism as Emerging Regulators of Dendritic Cell Function

**DOI:** 10.3389/fimmu.2019.02335

**Published:** 2019-09-27

**Authors:** Fabiola Osorio, Bart Everts

**Affiliations:** ^1^Immunology Program, Institute of Biomedical Sciences, Faculty of Medicine, University of Chile, Santiago, Chile; ^2^Leiden University Medical Center, Leiden, Netherlands

**Keywords:** dendritic cell, autophagy, epigenetics, metabolism, cellular stress, migration

As professional antigen presenting cells, Dendritic cells (DCs), undergo a well-defined activation process that render them competent to activate adaptive immune responses and also to control tolerance. As a result, DCs are considered key regulators of the immune system. DC activation via pathogen recognition or by tissue injury occurs by virtue of expression of pattern recognition receptors, which has been extensively documented in previous years. However, there is a growing body of evidence demonstrating that DC activation and function can be also finely adjusted by perturbations in cellular mechanisms that are normally associated with homeostasis, and that include processes such as cell polarity, changes in the secretory demand, endoplasmic reticulum (ER) stress, epigenetics, autophagy, and metabolism among others. Moreover, evidence is now emerging that environmental cues such as nutrient availability, antibody complexes, and sodium levels are also important regulators of DC biology. In this Research Topic, we have brought together a collection of 10 primary research and review papers from experts in their respective fields to home in novel and emerging regulators of DC function that go beyond canonical pattern recognition.

Several cellular “household” processes that normally function to maintain intracellular homeostasis can also serve as regulators of DC function and when changed or perturbed, may act as instigators of DC activation. In our Research Topic, five of these emerging processes are highlighted in a total of five reviews and three primary research articles. First, in a review by Münz, the most recent studies and evidence for a previously unappreciated role of macro-autophagy in regulating antigen processing and presentation are discussed. Second, Barbier et al. explore the role of myosin in actin remodeling, which in the case of DCs is important for migration. They performed a convincing set of experiments to show a key role for myosin II in maintaining fast cell speed specifically in confined microenvironments. Third, despite the relatively short lifespan of DCs, there is a growing body of evidence that suggests that epigenetic changes and chromatin remodeling are pivotal in determining DC fate and activation state. Boukhaled et al. delve into this topic and discuss in their review what signals control epigenetic changes in DCs and what the functional consequences are of such changes. Fourth, Medel et al. have investigated the relevance of ER stress in sensing tumor cell lysates by DCs. They showed that bone marrow derived DCs activate the unfolded protein response sensor IRE1 and the transcription factor XBP1s upon recognition of tumor cell lysates, which boosts proinflammatory cytokine production and cross-presentation of tumor cell-associated antigens to CD8+ T cells. Finally, currently one of the most rapidly developing immunological research areas is the field of immunemetabolism, which focuses on the role of cellular metabolism in shaping immune cell function. This concept has also permeated the DC field and there have been major recent advances in our understanding of the importance of central metabolic pathways and metabolic reprogramming in all aspects in DC biology. Four different contributions highlight the current status of, and provide exciting new insights in this field. Wculek et al. provide an insightful overview of how glucose, lipid, and amino acid metabolism shape DC differentiation and activation. In addition, they discuss the distinct metabolic programs in DCs that underlie induction of tolerance vs. immunity, as well as what the current evidence is for differences in metabolism between different DC subsets. In a study by Basit et al., this latter aspect is experimentally tested in different DC subsets from human blood. Their work reveals that CD1c+ and plasmacytoid DCs rely on glycolysis and oxidative phosphorylation for their activation, respectively, indicating that different DC subsets have very distinct metabolic requirements for their activation. In a timely review by Snyder and Amiel the most recent developments on the role of nutrient sensor mTOR in DC biology are discussed. From this article, it becomes clear that mTOR acts a central signaling hub that orchestrates the integration of nutrient and danger signals with DC metabolism, activation, and differentiation. Finally, given the growing appreciation that metabolic reprogramming plays a key role in DC differentiation, He et al. have dedicated a review on this specific topic. From their review, it becomes clear that the type metabolic programs involved in DC differentiation largely depend on the DC subset involved and whether it is studied *in vitro* or *in vivo*, or is still simply not known, illustrating that this field is still maturing.

One of the implications of the articles on metabolism and myosin, as highlighted in the previous section, is that nutrient availability in the micro-environment or the degree of spatial confinement that DCs reside in are important determinants of their function. However, there are other factors in the microenvironment, apart from canonical danger signals, that can have an equally significant impact on DCs. One such example is elegantly provided by Hoepel et al., who show that DCs when exposed to IgG immune complexes, in an FcγR and IRF5 dependent manner, boost pro-inflammatory cytokine production through cross-talk with Toll-like receptors. Likewise, it has become apparent that extracellular sodium can have a significant impact on immune cell function, including DCs. Neubert et al. review the recent literature on this topic and provide compelling examples of how local Na^+^ shapes DC function, but also how immune cells impact Na^+^ homeostasis, revealing that there is reciprocal regulation of DC biology and fluid homeostasis. This clearly illustrates that there is a wide variety of extrinsic cues DCs receive in a given microenvironment that go well beyond classical pattern recognition and that profoundly impact their function.

We thank all authors for their time and effort they put into their excellent contributions. Without their commitment this collection would not have been possible. We hope that you will enjoy reading the articles within this Frontiers collection, give you new insights into the factors that regulate DC function and spark new ideas. We believe that these studies and reviews are a testimony of the complex set of cell intrinsic and extrinsic cues that govern DC biology, but also demonstrate that we still have a long way to go before we have a complete understanding of the multilayered control of DC function.

## Author Contributions

All authors listed have made a substantial, direct and intellectual contribution to the work, and approved it for publication.

### Conflict of Interest

The authors declare that the research was conducted in the absence of any commercial or financial relationships that could be construed as a potential conflict of interest.

